# The assessment of depression awareness and help-seeking behaviour: experiences with the International Depression Literacy Survey

**DOI:** 10.1186/1471-244X-7-48

**Published:** 2007-09-13

**Authors:** Ian B Hickie AM, Tracey A Davenport, Georgina M Luscombe, Ye Rong, Megan L Hickie, Morag I Bell

**Affiliations:** 1 Brain & Mind Research Institute, The University of Sydney, Sydney, Australia; 2 Discipline of Psychological Medicine, The University of Sydney, Sydney, Australia

## Abstract

**Background:**

Depression causes substantial disease burden in both developed and developing countries. To reduce this burden, we need to promote understanding of depression as a major health condition. The International Depression Literacy Survey (IDLS) has been developed to assess understanding of depression in different cultural and health care settings.

**Methods:**

Four groups of Australian university students completed the survey: medical students in second (n = 103) and fourth (n = 82) years of a graduate course, ethnic Chinese students (n = 184) and general undergraduate students (n = 38).

**Results:**

Differences between the student groups were evident, with fourth year medical students demonstrating greater general health and depression literacy than second year medical students. Australian undergraduate students demonstrated better depression literacy than those from ethnic Chinese backgrounds. Ethnicity also influenced help seeking and treatment preferences (with more Chinese students being inclined to seek help from pharmacists), beliefs about discrimination and perceptions regarding stigma.

**Conclusion:**

The IDLS does detect significant differences in understanding of depression among groups from different ethnic backgrounds and between those who differ in terms of prior health training. These preliminary results suggest that it may be well suited for use in a wider international context. Further investigation of the utility of the IDLS is required before these results could be extrapolated to other populations.

## Background

Depressive disorders are a major source of non-fatal disease burden in developed countries, as well as being a key determinant of health-related disability in the developing world [1,2]. To achieve meaningful reductions in depression-related health burden a range of preventative and treatment strategies are urgently required [3,4]. The development of such initiatives, however, usually depends on broad recognition that depression is not only common and disabling but that it also responds to evidence-based treatments.

The concept of 'mental health literacy' has emerged to describe "*knowledge and beliefs about mental disorders which aid their recognition, management or prevention*" [5]. To date, assessment has rested largely on typical case-based vignettes of persons with schizophrenia or depression. This approach requires the respondent to identify the person as a 'case' of mental disorder and then disclose their knowledge or attitudes about available health services or treatments. This has proved to be a useful way of tracking community attitudes to a range of mental health problems and their treatments, particularly in English-speaking countries [6].

We propose an alternative approach which specifically describes depression as a health condition, and then seeks to determine understanding of its characteristics and impacts relative to other medical and psychological conditions. Through our social marketing strategies for *beyondblue: the national depression initiative *in Australia, we focused more on these general health and depression-specific literacy issues than on an individual's capacity to recognise a typical 'case' of mental disorder. Our earlier work indicated that while the general public was aware of the potential impacts of depression, common mental disorders generally were not seen to be as important as other major physical disorders such as cancer and heart disease [7]. Further, a range of other factors such as personal or family experiences of depression, previous experiences of seeking care or encountering other stigmatising attitudes towards persons with depression appeared to be impacting on willingness to seek mental health care.

As part of an international movement to promote greater recognition of the social and economic costs of depression [3,4], we were keen to develop this alternative approach to rating depression literacy. A priority was that the method should be able to be utilised easily in countries where English is not the first language or where there was little priority given to the provision of relevant health services. Consequently, we developed a modular depression literacy self-report instrument for use primarily in countries that are participating in the 'Reducing the Social and Economic Burdens of Depression' (SEBoD) movement in Asia.

As the SEBoD movement aims to improve depression literacy within general health workforces, we were interested to determine whether the instrument was sensitive to differences in mental health training. As many countries are not culturally homogeneous, we also sought to determine whether significant ethnic differences had impacts on depression literacy among persons residing in the same country. Therefore, in this Australian study we endeavoured to examine the utility of our instrument when it was completed by university students from different ethnic and health training backgrounds.

## Methods

The study was a cross-sectional survey of four groups of Australian university students. There were two groups of general students (ethnic Chinese and non-ethnically selected students) and two groups of medical students (second and fourth year students of The University of Sydney Graduate Medical Program).

### Study procedure

Students 18 years or older, who were enrolled in a university, were included in the study. The second and fourth year medical students were recruited during seminar series devoted to the teaching of mental health topics. The fourth year students were engaged in the clinical aspects of psychiatry and addiction medicine. Ethnic Chinese students were contacted and invited to the study through Chinese student organisations. The survey was also distributed to students attending a college of The Australian National University (ANU, Canberra) using a convenience sampling approach. The study was approved by The University of Sydney Human Research Ethics Committee.

### Instrument

Version 1.1 of the survey was divided into seven sections [see Additional file 1]. At the end of each section, subjects are given the option of continuing the survey or terminating. The survey covers a range of topics, including:

#### Section A

##### Demographics

This section includes questions on ethnicity, education and identity in the health care system.

#### Section B

##### Major health problems

In order to determine the salience of mental health disorders, and depression in particular, as health issues, respondents are requested to nominate the main causes of death or disability in their country from a list of general health problem categories, a list of specific illness and injuries and a list of mental health problems. These lists were based on the top 13 'health problems' and the top 23 'diseases' causing the most death or disability in Disability Adjusted Life Years (DALYs) in the world as determined by World Health Organization [8], and the mental and behavioural disorder sub-categories listed in the International Classification of Diseases [ICD-10; 9].

##### Knowledge of depression

Respondents nominate from lists what they believe are the most typical symptoms and common experiences with depression [10], and the prevalence of depression.

#### Section C

##### Help and treatment

These questions are based on research about treatments for depression previously conducted in Australia [11,12]. Respondents are asked their opinion about the most likely outcome of depression with or without professional help. The likelihood of seeking help from professionals or non-professionals and level of helpfulness of various treatments are also rated using a five-point Likert-type scale. Personal experience with depression is included in this section.

#### Section D

##### Information

This question identifies experiences in seeking information for depression.

#### Section E

##### Perceived needs

The General-practice Users' Perceived-need Inventory (GUPI) is used to identify perceived need for mental health care provided by general practitioners (GPs) [13]. The questions ask about personal willingness to discuss emotional problems with GPs and any reasons stopping the individual doing so. This section only applies to subjects who sought help from a GP for an emotional problem during the 12 months prior to their participation in the study.

#### Section F

##### Attitudes to depression and its treatment

The first question about discrimination was taken from previous Australian research on attitudes to depression [7]. The second item asks about attitudes towards people with "*severe depression*" and was derived from research conducted in the United Kingdom investigating the stigma associated with mental health problems [14].

#### Section G

##### General information

The Kessler Psychological Distress Scale (K10) [15] is included in this section to measure current levels of psychological distress. The 12-item Somatic and Psychological HEalth REport (SPHERE) [16] is used to assess the severity of psychological and/or somatic symptoms. Two disability questions follow [17]. Two further demographic questions obtain information on living and employment conditions.

### Development of the survey

The survey utilised the concepts of knowledge, awareness and attitudes that have been more generally used in mental health literacy research and the evaluation of other mental health promotion campaigns [6]. However, it made additional use of those items that were developed to assist with the assessment of the impact of *beyondblue *in Australia [12]. Additional elements drew on previous research assessing the impacts of stigma in relation to persons with mental disorders [14] as well as the specific development of a brief questionnaire to assess experiences of mental health service use [13].

As the aim was to develop a basic self-report format for use in the countries and cultures of the Asia Pacific Region, a draft survey was sent to SEBoD committee members and country chapter heads. They were asked to comment particularly on the suitability and comprehensibility of the survey in their local circumstances. The draft was modified according to their comments and suggestions. The items were simplified and pre-tested among a group of native English speakers to ensure the survey was understandable for people with various levels of English literacy. It was also tested and commented upon by culturally diverse community members, including Chinese, Japanese and Korean, for cultural appropriateness.

### Statistical analysis

For the items regarding major health problems, specific illness or injuries and mental health problems, only those categories nominated by over 10% of students are reported. Additionally, only those categories nominated by over 20% of students within the items concerning typical signs, behaviours and experiences of persons with depression are reported in this article. For the questions on help seeking, only the combined percentages of the answers "probably likely" and "definitely likely" are presented. Likewise, only the percentages for combined positive answers are described, such as "probably likely" and "definitely likely" for the discrimination item, and "agree" and "strongly agree" for the stigma item. These combined categories were also used for the purposes of statistical analysis.

For the K10, the response options "none of the time", "a little of the time", "some of the time", "most of the time" and "all of the time" were coded as 1, 2, 3, 4 and 5 respectively. The sum of the scores for each item were calculated and recoded into three levels of psychological distress: 10–15 representing low or no, 16–29 representing medium, and 30–50 representing high distress. The last two categories were combined both for the purposes of presentation and for statistical analysis.

The response options in the SPHERE questionnaire, "never or some of the time", "a good part of the time" and "most of the time" were coded as 0, 1, and 2 respectively. The total scores of the six psychological questions and the six somatic questions were calculated separately. The students with a total score of two or more for the six psychological items were categorised as having strong psychological symptoms (PSYCH-6); the students with a total score of three or more for the six somatic items were categorised as having strong somatic symptoms [16]. The percentages of students who showed significant psychological and/or somatic symptoms were reported and compared among the groups.

Two sets of analyses were conducted, the first comparing second year medical students with fourth year medical students (to examine the effect of mental health training), the second comparing Chinese students with ANU students (to examine the effect of culture). Associations between group membership (type of student) and item responses were made using the Chi-square test, the continuity correction value being considered for two by two tables. Age was significantly skewed, and this could not be rectified, so the non-parametric Mann-Whitney U test was used for the two group comparison. All calculations and analyses were carried out using the Statistical Package for the Social Sciences (SPSS) version 12. Where appropriate, Bonferroni corrections were applied to alpha to account for multiple comparisons.

## Results

The English version of the International Depression Literacy Survey (IDLS) was administered to four student groups: medical students from The University of Sydney in second year (n = 103) and fourth year (n = 82); tertiary students from The ANU (n = 38); and Chinese students from The University of Sydney, The University of Technology (Sydney) and The University of New South Wales (n = 184). In total the survey was completed by N = 407 students. Eighty-two percent of the sample completed the entire survey (n = 333/407), 33 terminated after section B (i.e. only completed the first two sections), 17 after section C, 12 after section D, six after section E and six after section F.

### Demographics

The mean age of students was 24.4 years (SD = 4.0 years; range = 15 – 43 years), with Chinese students being significantly older than ANU students, and fourth year medical students significantly older than second year students (see Table 1). A small proportion of the ANU students had a medical/health care background (8%), as did the ethnic Chinese students (9%). Conversely, a small proportion of medical students reported Chinese nationality (second year: 4%, fourth year: 1%), as did the ANU students (3%).

**Table 1:** Demographic characteristics of sample (N = 407)

	**2^nd ^Year Medical**	**4^th ^Year Medical**	**ANU**	**Chinese**	**2^nd ^vs 4^th ^Year Medical**	**ANU vs Chinese**
			
	n = 103*	n = 82*	n = 38*	n = 184*	**p value**	**p value**
Age (years): mean (SD)	25.5 (4.0)	27.5 (3.2)	20.3 (2.1)	23.3 (3.5)	< 0.001^1^	< 0.001^1^
Age (years): range	21 – 42	23 – 43	18 – 26	15 – 39		
% female	55	59	47	46	0.693	1.000
Area inhabited: urban, %	97	99	82	75	0.819	0.502

^1 ^Mann-Whitney U test.

*There are missing data for different items (age, gender: 2^nd ^year medical n = 1, 4^th ^year medical n = 4, ANU none missing, Chinese n = 8 for age and n = 1 for gender; area inhabited: 2^nd ^year medical none missing, 4^th ^year medical n = 4, ANU none missing, Chinese n = 5).

### Major health problems

There were significant differences between the various student groups with regard to the recognition of major health problems in Australia (see Table 2). For example, approximately half of the Chinese students nominated "*heart disease and stroke*" as a main cause of death or disability, compared to the majority of ANU students. By contrast for "*brain, behavioural and mental health disorders*", just over half of the ANU students rated this key area as a major health problem compared with only a quarter of ethnic Chinese students.

**Table 2:** Major health problems students nominated as the main causes of death or disability in Australia (N = 382*)

	**2^nd ^Year Medical**	**4^th ^Year Medical**	**ANU**	**Chinese**	**2^nd ^vs 4^th ^Year Medical**	**ANU vs Chinese**
			
	*n = 101*	*n = 81*	*n = 38*	*n = 162*	p value^a^	p value^a^
Cancer %	77	56	84	85	0.003	1.000
Heart disease and stroke %	96	91	92	49	0.315	< 0.001
Brain, behavioural and mental health disorders %	61	85	53	24	0.001	0.001
Diabetes %	25	36	37	51	0.145	0.177
Lung and chest disease %	56	52	16	15	0.640	1.000
Accidental injuries %	36	10	37	28	< 0.001	0.409
Infectious disease %	8	0	3	33	0.026	< 0.001
Muscle or joint diseases %	10	40	5	4	< 0.001	1.000
Non-accidental injuries %	2	4	5	22	0.802	0.030

^a^Adjusted alpha = 0.05/9 = 0.006.

* Data were missing for 6% of the valid sample (n = 25/407).

There also appeared to be a training effect, with significantly more fourth year medical students nominating "*brain, behavioural and mental health disorders*" as a major health problem than second year medical students.

When asked about specific illnesses and injuries, rather than broad disease categories, a similar pattern of responses was observed with significant group differences observed in both sets of analyses (see Table 3). For example, just over half of the Chinese students nominated "*heart attack or other heart disease*" as a main cause of death or disability, compared to more than 90% of ANU students. There was a trend towards a difference between the medical student groups in nominating "*depression*" as a specific illness causing death or disability, the proportion increasing with mental health training.

**Table 3:** Specific illnesses or injuries students nominated as the main causes of death or disability in Australia (N = 380*)

	**2^nd ^Year Medical**	**4^th ^Year Medical**	**ANU**	**Chinese**	**2^nd ^vs 4^th ^Year Medical**	**ANU vs Chinese**
	
	*n = 103*	*n = 81*	*n = 37*	*n = 159*	p value^a^	p value^a^
Heart attack or other heart disease %	93	91	92	57	0.850	< 0.001
Depression %	67	84	30	19	0.014	0.215
Stroke or other brain disease %	71	53	46	24	0.020	0.013
Diabetes %	31	63	38	45	< 0.001	0.523
Lung cancer %	39	14	73	42	< 0.001	0.001
Road traffic accidents %	49	16	35	38	< 0.001	0.860
Alcohol abuse %	28	46	27	34	0.021	0.538
Alzheimer's disease or other dementias %	22	22	19	21	1.000	0.914
Asthma %	20	22	11	21	0.904	0.246
Osteoarthritis %	22	44	16	3	0.002	0.003
HIV infection or AIDS %	3	0	8	38	0.336	0.001
Colon or rectum (bowel) cancer %	18	12	16	16	0.450	1.000
Suicide or self-harm %	3	11	8	25	0.053	0.042
Emphysema or chronic bronchitis %	28	20	0	7	0.253	0.211
Lung or other chest infections %	31	7	5	9	< 0.001	0.729

^a ^Adjusted alpha = 0.05/15 = 0.003.

* Data were missing for 7% of the valid sample (n = 27/407).

#### Mental health problems

For the specific question related to mental health problems, differences with regard to depression recognition were apparent (see Table 4). The highest proportion nominating "*depressive illness*" was in the fourth year medical student group (94%), ranging down to only 17% for the ethnic Chinese students. However, the responses from the medical students were very similar. The proportions of ANU students nominating "*depressive illness*" and "*alcohol abuse or addiction*" were significantly higher than in the Chinese student group, but the situation was reversed when considering "*anxiety, neurosis or panic disorder*".

**Table 4:** Mental health problems students nominated as the main causes of death or disability in Australia (N = 384*)

	**2^nd ^Year Medical**	**4^th ^Year Medical**	**ANU**	**Chinese**	**2^nd ^vs 4^th ^Year Medical**	**ANU vs Chinese**
			
	*n = 103*	*n = 82*	*n = 37*	*n = 162*	p value^a^	p value^a^
Depressive illness %	86	94	51	17	0.154	< 0.001
Drug abuse or addiction %	25	33	62	46	0.325	0.104
Alcohol abuse or addiction %	51	61	49	18	0.202	< 0.001
Dementia, Alzheimer's disease or other brain damage %	45	44	27	34	1.000	0.538
Schizophrenia or other psychoses %	29	28	16	34	1.000	0.056
Anxiety, neurosis or panic disorder %	23	18	11	41	0.517	0.001
Eating disorder %	12	6	22	19	0.297	0.840
Manic depressive illness %	11	2	22	18	0.059	0.771

^a ^Adjusted alpha = 0.05/8 = 0.006.

* Data were missing for 6% of the valid sample (n = 23/407).

### Perceptions regarding people with depression

There was a substantial degree of variation in recognition of common symptoms of depression (see Table 5). A significantly greater proportion of ANU students than Chinese students recognised "*feeling overwhelmed*" as a typical sign of depression. It is of interest to note that there were also some shifts in responses between the second and fourth year medical students, for instance a greater recognition following mental health training of fatigue as a typical symptom of depression.

**Table 5:** Signs or symptoms typifying a person with depression as nominated by Australian students (N = 394*)

	**2^nd ^Year Medical**	**4^th ^Year Medical**	**ANU**	**Chinese**	**2^nd ^vs 4^th ^Year Medical**	**ANU vs Chinese**
			
	*n = 103*	*n = 82*	*n = 37*	*n = 172*	p value^a^	p value^a^
Being sad, down or miserable %	71	82	46	50	0.125	0.790
Sleep disturbance %	60	79	38	21	0.009	0.048
Being unhappy or depressed %	54	55	35	31	1.000	0.750
Feeling tired all the time %	31	55	38	24	0.002	0.121
Thinking "Life is not worth living" %	33	29	35	32	0.700	0.858
Thinking "I'm worthless" %	40	34	19	29	0.524	0.292
Thinking "I'm a failure" %	43	11	24	31	< 0.001	0.514
Having no confidence %	29	16	32	29	0.051	0.835
Feeling frustrated %	7	1	22	37	0.137	0.105
Feeling overwhelmed %	28	13	51	11	0.025	< 0.001

^a ^Adjusted alpha = 0.05/10 = 0.005.

* Data were missing for 3% of the valid sample (n = 13/407).

### Common behaviours and experiences

When asked about the typical behaviours and experiences of people with depression, there were some differences among the student groups (see Table 6). Most notably, withdrawal was recognised as a typical behaviour by over two-thirds of the ANU students, but less than a third of the Chinese students. Otherwise the responses in these two groups were largely similar. In terms of possible training effects, the only significant difference between the medical student groups was for "*lack of self care*", fewer fourth year students nominating this behaviour. Suicidal ideation or behaviour was also less frequently nominated as typical of people with depression following training.

**Table 6:** Typical behaviours and experiences of people with depression as nominated by Australian students (N = 394*)

	**2^nd ^Year Medical**	**4^th ^Year Medical**	**ANU**	**Chinese**	**2^nd ^vs 4^th ^Year Medical**	**ANU vs Chinese**
			
	*n = 103*	*n = 82*	*n = 37*	*n = 172*	p value^a^	p value^a^
Be unable to concentrate or have difficulty thinking %	62	78	41	35	0.030	0.644
Stop doing things they enjoy %	63	65	32	18	0.952	0.081
Withdraw from close family and friends %	52	42	70	28	0.228	< 0.001
Have relationship or family *problems *%	37	44	27	34	0.415	0.509
Stop going out %	38	39	32	26	0.993	0.566
Become dependent on alcohol, drugs or sedatives %	19	37	41	38	0.014	0.900
Have suicidal thoughts or behaviours %	46	26	35	29	0.008	0.595
Not get things done at school/work %	28	38	35	26	0.217	0.366
Lack of self care (e.g. have a change in their personal hygiene habits) %	33	12	14	21	0.002	0.422

^a^Adjusted alpha = 0.05/9 = 0.006.

* Data were missing for 3% of the valid sample (n = 13/407).

When asked to estimate the proportion of people who will experience depression in their lives, there was a significant difference between the non-medical student groups in the proportion correctly nominating "one in five" (51% of the ANU students and 23% of the Chinese students), but not between the medical students (49% second year and 60% of fourth year students; see Table 7).

**Table 7:** Students perceptions of the approximate proportion of people who experience depression at some point in their lives (N = 391*)

	**2^nd ^Year Medical**	**4^th ^Year Medical**	**ANU**	**Chinese**	**2^nd ^vs 4^th ^Year Medical**	**ANU vs Chinese**
			
	*n = 103*	*n = 82*	*n = 37*	*n = 169*	p value	p value
One in 50 people %	4	1	11	17		
One in 20 people %	18	13	22	27		
One in 10 people %	28	26	16	22		
One in 5 people %	49	60	51	23	0.171	0.001
Don't know %	1	0	0	11		

* Data were missing for 4% of the valid sample (n = 16/407).

### Help seeking

Students were asked to rate how likely they would be to seek help from a list of health professionals (see Table 8). There were no significant differences between the medical student groups. The most notable difference between the ANU and ethnic Chinese students was the proportion reporting they would be probably or definitely likely to seek help from a pharmacist, the proportion being seven times higher in the Chinese group.

**Table 8:** Proportion of Australian students who reported being probably or definitely likely to seek professional help if they thought they were experiencing depression (N = 345*)

	**2^nd ^Year Medical**	**4^th ^Year Medical**	**ANU**	**Chinese**	**2^nd ^vs 4^th ^Year Medical**	**ANU vs Chinese**
			
	*n = 90*	*n = 78*	*n = 37*	*n = 140*	p value^a^	p value^a^
Counsellor %	54	46	53	58	0.359	0.719
General or family doctor %	82	89	47	67	0.358	0.048
Pharmacist %	8	3	6	43	0.250	< 0.001
Psychiatrist %	51	65	43	55	0.085	0.301
Psychologist %	59	56	44	69	0.897	0.011
Social worker %	11	11	31	48	1.000	0.092
Welfare officer %	2	1	26	42	1.000	0.115
No one/wouldn't seek help %	32	33	47	42	1.000	0.790

^a ^Adjusted alpha = 0.05/8 = 0.006.

* Data were missing for 8% of the valid sample (n = 29/374).

Students were then asked about seeking help from non-professional sources. Again, there were no significant differences between second and fourth year medical students. A greater proportion of Chinese students than ANU students indicated they were likely to seek help from non-professionals (such as acupuncturists, religious persons, naturopaths or herbalists, personal trainers and traditional healers; see Table 9).

**Table 9:** Proportion of Australian students who reported being probably or definitely likely to seek non-professional help if they thought they were experiencing depression (N = 350*)

	**2^nd ^Year Medical**	**4^th ^Year Medical**	**ANU**	**Chinese**	**2^nd ^vs 4^th ^Year Medical**	**ANU vs Chinese**
			
	*n = 91*	*n = 78*	*n = 37*	*n = 144*	p value^a^	p value^a^
Acupuncturist %	5	4	6	34	1.000	0.002
Clergy, priest or other religious person %	23	14	11	44	0.235	0.001
Family %	89	87	71	84	0.873	0.140
Friends %	88	87	83	88	1.000	0.659
Naturopath or herbalist %	9	5	11	42	0.522	0.002
Personal trainer, exercise manager or relaxation instructor %	26	32	19	55	0.504	< 0.001
Traditional healer %	7	3	6	35	0.369	0.001

^a ^Adjusted alpha = 0.05/7 = 0.007.

* Data were missing for 6% of the valid sample (n = 24/374).

Students were asked to rate the perceived helpfulness or harmfulness of various common treatments for depression (see Table 10). There were no significant differences between the medical students in terms of the proportion nominating treatments as helpful. However, differences emerged between the non-medical students (ANU and ethnic Chinese). Over two-thirds of the Chinese students (72%) rated "*reading self-help books*" as helpful, compared with only 39% of ANU students. More ANU students (70%) thought antidepressant medications would be helpful than did Chinese students (46%). Similarly, there was trend towards a difference regarding the helpfulness of "*having an occasional alcoholic drink*", with 33% of Chinese students considering this helpful, compared with only 13% of ANU students.

**Table 10:** Students perceptions of the helpfulness of specific treatments for depression (N = 342*)

	**2^nd ^Year Medical**	**4^th ^Year Medical**	**ANU**	**Chinese**	**2^nd ^vs 4^th ^Year Medical**	**ANU vs Chinese**
			
	*n = 91*	*n = 78*	*n = 36*	*n = 133*	p value^a^	p value^a^
Becoming more physically active %	99	100	97	84	1.000	0.063
Changing your diet %	71	55	71	63	0.069	0.547
Having an occasional alcoholic drink %	17	21	13	33	0.676	0.044
Reading about people with similar problems and how they have dealt with them %	85	88	65	75	0.796	0.303
Reading self-help book(s) %	59	69	39	72	0.273	0.001
Taking antidepressant medications %	92	99	70	46	0.115	0.029
Taking natural remedies (e.g. vitamins) %	26	28	42	51	0.992	0.500
Taking sleeping tablets or sedatives %	24	12	14	31	0.092	0.117
Using brief counselling therapies (e.g. cognitive and/or behavioural therapies) %	100	96	82	68	0.184	0.178
Using long-term counselling %	94	87	77	66	0.156	0.352

^a ^Adjusted alpha = 0.05/10 = 0.005.

* Data were missing for 9% of the valid sample (n = 32/374).

### Discrimination and stigma

When asked about the perceived likelihood of discrimination if they, or someone close to them, experienced depression, the only difference in response amongst the medical students was regarding "*a government or other public welfare agency*", with a quarter of second year medical students believing this was definitely or probably likely, compared with 42% of fourth year medical students (see Table 11). There was greater disparity in the perceived likelihood of discrimination between the non-medical groups. Only 7% of ANU students felt that discrimination was definitely or probably likely within the context of a public or private hospital, compared with 44% of Chinese students. Similarly, only 4% of ANU students felt that discrimination was likely from a "*doctor or other health professional*" compared with 37% of the Chinese students.

**Table 11:** Proportion of Australian students who reported discrimination was probably or definitely likely from various persons or organisations if they, or someone close to them was experiencing depression (N = 298*)

	**2^nd ^Year Medical**	**4^th ^Year Medical**	**ANU**	**Chinese**	**2^nd ^vs 4^th ^Year Medical**	**ANU vs Chinese**
			
	*n = 69*	*n = 70*	*n = 29*	*n = 130*	p value^a^	p value^a^
A bank, insurance company or other financial institution %	42	50	54	44	0.426	0.476
A government or other public welfare agency %	25	42	19	39	0.049	0.073
A public or private hospital %	17	25	7	44	0.308	0.001
Other people who don't know you well %	94	88	89	66	0.379	0.048
Your doctor or other health professional %	7	18	4	37	0.120	0.002
Your employer %	72	77	67	67	0.621	1.000
Your family %	18	19	21	42	1.000	0.052
Your friends %	24	22	21	43	1.000	0.042

^a ^Adjusted alpha = 0.05/8 = 0.006.

* Data were missing for 12% of the valid sample (n = 41/339).

Students were asked whether they agreed or disagreed with statements concerning people with severe depression (see Table 12). There were no significant differences between attitudes towards people with severe depression between the medical student groups. In contrast, 48% of Chinese students agreed with the statement "*have themselves to blame*", compared with 7% of ANU students; and 39% of Chinese students agreed with the statement that people with severe depression "*shouldn't have children in case they pass on the illness*" compared with none of the ANU students.

**Table 12:** Proportion of Australian students who agreed or strongly agreed with statements regarding people with severe depression (N = 298*)

**People with depression...**	**2^nd ^Year Medical**	**4^th ^Year Medical**	**ANU**	**Chinese**	**2^nd ^vs 4^th ^Year Medical**	**ANU vs Chinese**
			
	*n = 70*	*n = 70*	*n = 30*	*n = 128*	p value^a^	p value^a^
Are dangerous to others %	5	3	22	37	0.987	0.222
Are hard to talk to %	72	66	60	68	0.554	0.668
Are often artistic or creative people when they are well %	53	45	65	54	0.583	0.564
Are often very productive people when they are well %	94	90	80	55	0.690	0.068
Have themselves to blame %	6	1	7	48	0.362	< 0.001
Often make good employees when they are well %	93	90	77	60	0.843	0.316
Often perform poorly as parents %	51	45	42	53	0.650	0.460
Often try even harder to contribute to their families or work when they are well %	77	81	77	64	0.879	0.371
Shouldn't have children in case they pass on the illness %	0	1	0	39	1.000	< 0.001
Should pull themselves together %	16	18	23	41	0.975	0.143

^a ^Adjusted alpha = 0.05/10 = 0.005.

* Data were missing for 12% of the valid sample (n = 41/339).

### K10 and SPHERE

As shown in Table 13, more than half of the students were experiencing medium or high levels of psychological distress (as measured by the K10) during the 30 days prior to participation in the study.

**Table 13:** Levels of current psychological distress as measured by the K10 and the SPHERE (N = 309*)

		**2^nd ^Year Medical**	**4^th ^Year Medical**	**ANU**	**Chinese**	**2^nd ^vs 4^th ^Year Medical**	**ANU vs Chinese**
				
		*n = 77*	*n = 71*	*n = 35*	*n = 126*	**p value**	**p value**
**K10**	low or no psychological distress %	48	52	55	24		
	medium or high psychological distress %	53	48	45	76	0.687	0.002
**SPHERE**	Any PSYCH-6 disorder %	31	25	26	56	0.547	0.003
	Any SOMA-6 disorder %	26	20	46	56	0.450	0.355

K10 = Kessler Psychological Distress scale, SPHERE = Somatic and Psychological HEalth REport, PSYCH-6 disorder = a total score of two or more for the six SPHERE psychological items indicating strong psychological symptoms, SOMA-6 = a total score of three or more for the six SPHERE somatic items indicating strong somatic symptoms.

* Data were missing for 7% of the valid sample (n = 24/333).

A significantly greater proportion of the Chinese students showed medium or high levels of psychological distress on the K10 than the ANU students, whereas there was no difference between the medical student groups. Consistent with this, the Chinese students indicated experiencing substantial psychological and somatic symptoms as measured by the SPHERE, with substantial psychological symptoms being significantly more prevalent than in the ANU students.

## Discussion

The IDLS has been developed specifically to track understanding of depression as a general health condition. For the IDLS to be useful within ethnically diverse communities or groups of health professionals over time, it should first be sensitive to likely baseline differences between relevant groups residing in the same country. While there is little published research regarding the specifics of cross-cultural differences regarding attitudes and beliefs around depression, studies published to date suggest attitudes to mental illness in general differ between Asian and western cultures [18,19]. The study reported here demonstrates that the IDLS does detect clear differences between medical students in second and fourth year courses, and between non-medical students from ethnic Chinese backgrounds and other undergraduates residing in Australia. These differences are obvious across all the key areas (depression within the context of major general health problems and major mental health problems, common psychological symptoms of depression, attitudes to the use of evidence-based treatments, patterns of health care utilisation and expectations of discrimination).

The basic utility of the instrument for potential use in wider epidemiological, cross-cultural, longitudinal or interventional studies is given preliminary support by the current results. However, it must be noted that convenience samples were utilised in this study, thus limiting the conclusions that can be drawn. The effect of health training on responses would ideally be confirmed by a longitudinal study, sampling the same individuals prior to and following mental health training. Another limitation was the age difference within each of the comparison student groups. While there did not appear to be a systematic effect with increasing age, the study would warrant repetition in age matched samples. While we acknowledge that a small proportion of the Chinese students had a medical or health care background, and that a small proportion of the other students (medical or from ANU) were Chinese, this would most probably reduce the likelihood of finding differences attributable to culture or training. As the instrument has been designed in consultation with professional leaders in a wide range of Asia-Pacific countries, we now encourage utilisation of at least the English version of the instrument in relevant studies among English-literate groups within those countries. A second set of studies will now address the utility of translated versions of the instrument for use in other population groups not literate in English within those countries.

Within the Australia setting, the differences detected between the student groups examined are perhaps unexpected and worthy of further comment. Specific health care training clearly has an association with increasing recognition of the general health burden due to depression. This association also appears to increase with the degree of that training and exposure to direct clinical experience of mental disorders (i.e. fourth versus second year medical students). The importance of medical courses increasing their commitment to this style of teaching in Australia and across the region needs to be emphasised [20,21]. Secondly, those students from ethnic Chinese backgrounds in Australia have markedly different knowledge and attitudes towards depression. This does not simply reflect an unwillingness to discuss such difficulties as the relevant self-report instruments indicate moderately high levels of psychological distress among this cohort. The lack of recognition of depression as a general health problem, the tendency to recognise features more typically conceptualised as anxiety, the greater reliance on pharmacists and alternative practitioners rather than traditional medical services and the expectation of greater discrimination all appear to be consistent with attitudes expressed within other ethnic Chinese groups not residing in Australia [22,23]. Such effects have major implications for current public awareness and health literacy interventions in Australia [4,7]. While we are recording substantial shifts in public attitudes among the broad Australian public [12], we may need to deal more effectively with those specific attitudes held by key ethnic groups or those who have recently arrived in our country.

## Conclusion

The IDLS does detect significant differences in understanding of depression among groups from different ethnic backgrounds and between those who differ in terms of prior health training. These results provide preliminary support for the suitability of the IDLS for use in a wider international context.

## Abbreviations

ANU- The Australian National University; 

DALYs- Disability Adjusted Life Years; 

GUPI- General-practice Users' Perceived-need Inventory; 

IDLS-  International Depression Literacy Survey; 

SEBoD-  "Reducing the Social and Economic Burdens of Depression" in Asia; 

SPHERE-  Somatic and Psychological HEalth Report.

## Competing interests

The author(s) declare that they have no competing interests.

## Authors' contributions

IBH designed the survey and drafted the manuscript. TAD participated in the survey design and drafting the manuscript. GML participated in the survey design, performed the statistical analysis and participated in drafting the manuscript. YR participated in carrying out the study surveys and drafting the manuscript. MLH participated in carrying out and coordination of the study. MIB participated in statistical analysis and drafting the manuscript.

All authors read and approved the final manuscript.

## Pre-publication history

The pre-publication history for this paper can be accessed here:



## Supplementary Material

Additional file 1 IDLS vn1.2.pdf. Version 1.2 of the International Depression Literacy Survey (IDLS) is linked to this manuscript. The authors encourage the use of the instrument, and would appreciate your feedback (contact Professor Ian Hickie; ianh@med.usyd.edu.au). Click here for file
