# Supplementary material for: The assessment of depression awareness and help-seeking behaviour: experiences with the International Depression Literacy Survey
Source: BMC Psychiatry. 2007 Sep 13;7:48. doi: 10.1186/1471-244X-7-48 (PMC2080627; doi:10.1186/1471-244X-7-48)
Supplement: Additional file 1 — IDLS vn1.2.pdf. Version 1.2 of the International Depression Literacy Survey (IDLS) is linked to this manuscript. The authors encourage the use of the instrument, and would appreciate your feedback (contact Professor Ian Hickie; ianh@med.usyd.edu.au). [file 1471-244X-7-48-S1.pdf]

# AN INTERNATIONAL HEALTH SURVEY

brain&mind RESEARCH INSTITUTE

**This survey is completely anonymous and should only take about 15 to 20 minutes to finish.**

**You will be asked questions about your views on the general health and well being of people in the country where you currently live. It is not a test, so there are no right or wrong answers.**

**Your views will help to inform local health policy, research and education programs.**

## A. DEMOGRAPHICS

Q1. Your age: ☐☐ years

Q2. Gender: ☐ Male ☐ Female

Q3a. What is your nationality?____________________

Q3b. Which language do you usually speak at home?

________________________________________

Q4a. Which country are you currently living in?

________________________________________

Q4b. Have you lived in that country for more than 12 months?

☐ Yes ☐ No

Q5a. Do you live in a rural, regional or urban area?

☐ Rural ☐ Urban
☐ Regional

Q5b. What is your area, post or zip code? ____________

Q6. What is your highest level of education?

☐ No formal education
☐ Completed or partially completed primary education
☐ Completed or partially completed secondary education
☐ Completed or partially completed tertiary education (e.g. college, university)
☐ Other (please specify): ____________________

Q7. Have you ever lived or studied outside the country in which you now live?

☐ Yes ☐ No

Q8. Are you currently:
(you can mark more than 1 answer)

☐ A patient, a consumer of health services or a person with an illness
☐ A carer or family member of a person with an illness
☐ A health professional (e.g. a doctor, nurse, psychologist, pharmacist)
☐ None of the above (please specify):______________
________________________________________

**If you are NOT a health professional, please go to Section B.**

Q9a. If you are a health professional, in what area do you practice?

☐ General or family doctor
☐ Pharmacist
☐ Psychologist
☐ Counsellor
☐ Nurse
☐ Psychiatrist
☐ Medical specialist (not a psychiatrist)
☐ Traditional health worker (e.g. herbalist, acupuncturist)
☐ Other (please specify):____________________

Q9b. If you are a health professional, how many years have you been in clinical practice?

☐☐ years

Q9c. If you are a health professional, have you ever studied or practiced outside the country in which you now live?

☐ Yes ☐ No

**Thinking of the country you now live in, please answer the following questions.**

## B. MAJOR HEALTH PROBLEMS

Q10. Right now, what do you think are the main causes of death or disability in this country (mark up to 4 answers)?

- ☐ Cancer (e.g. lung, liver, breast)
- ☐ Lung and chest diseases (e.g. asthma, emphysema)
- ☐ Heart disease and stroke
- ☐ Stomach, bowel and liver disease (e.g. stomach ulcer, cirrhosis of liver)
- ☐ Infectious diseases (e.g. HIV/ AIDS, diarrhoea, tuberculosis)
- ☐ Non-accidental injuries (e.g. self-inflicted, suicide, violence, war)
- ☐ Complications of pregnancy or childbirth
- ☐ Muscle or joint diseases (e.g. arthritis)
- ☐ Diabetes (high blood sugar)
- ☐ Brain, behavioural and mental health disorders (e.g. depression, alcohol and drug abuse, dementia, manic-depressive illness, schizophrenia, anxiety, neurasthenia)
- ☐ Lung and chest infections (e.g. pneumonia)
- ☐ Accidental injuries (e.g. road traffic accidents, falls)
- ☐ Vision or hearing impairment or loss
- ☐ Other (please specify):______________________________

Q11. Right now, which of these specific ILLNESSES or INJURIES cause the most death or disability in this country (mark up to 6 answers)?

- ☐ HIV infection or AIDS
- ☐ Lung cancer
- ☐ Measles
- ☐ Osteoarthritis
- ☐ Lung or other chest infections
- ☐ Road traffic accidents
- ☐ Schizophrenia
- ☐ Stomach cancer
- ☐ Stroke or other brain disease
- ☐ Suicide or self-harm
- ☐ Tuberculosis
- ☐ Vision impairment or loss
- ☐ Alzheimer's disease or other dementias
- ☐ Alcohol abuse
- ☐ Asthma
- ☐ Cataracts
- ☐ Colon or rectum (bowel) cancer
- ☐ Depression
- ☐ Diabetes
- ☐ Diarrhoea or dysentery
- ☐ Emphysema or chronic bronchitis
- ☐ Hearing impairment or loss
- ☐ Heart attack or other heart disease
- ☐ Other (please specify):______________________________

Q12. Right now, which MENTAL HEALTH problems cause the most death or disability in this country (mark up to 3 answers)?

- ☐ Schizophrenia or other psychoses
- ☐ Personality disorders
- ☐ Mental retardation or intellectual disorders
- ☐ Anxiety, neurosis or panic disorder (e.g. neurasthenia)
- ☐ Depressive illness
- ☐ Drug abuse or addiction
- ☐ Adolescent behavioural or emotional disorders
- ☐ Dementia, Alzheimer's disease or other brain damage
- ☐ Eating disorders (e.g. anorexia nervosa, bulimia nervosa, severe obesity)
- ☐ Alcohol abuse or addiction
- ☐ Manic depressive illness (or bipolar disorder)
- ☐ Don't know
- ☐ Other (please specify):_______________________

Q13a. Which of the following are the most typical of a person with depression (mark up to 5 answers)?

- ☐ Being sad, down or miserable
- ☐ Feeling frustrated
- ☐ An upset stomach
- ☐ Feeling overwhelmed
- ☐ Thinking "It's all my fault"
- ☐ Feeling disappointed
- ☐ Being irritable or cranky
- ☐ Thinking "I'm a failure"
- ☐ Weight loss
- ☐ Feeling guilty
- ☐ Sleep disturbance
- ☐ Headaches and muscle pains
- ☐ Thinking "Life is not worth living"
- ☐ Having no confidence
- ☐ Feeling tired all the time
- ☐ Poor appetite
- ☐ Thinking "I'm worthless"
- ☐ Feeling sick and run down
- ☐ Being unhappy or depressed
- ☐ Thinking "Nothing good ever happens to me"
- ☐ Being indecisive
- ☐ Don't know
- ☐ Other (please specify):_______________________

Q13b. Which of the following are people with depression most likely to do or have happen to them (mark up to 4 answers)?

- ☐ Be unable to concentrate or have difficulty thinking
- ☐ Not get things done at school/ work
- ☐ Experience discrimination
- ☐ Stop going out
- ☐ Have relationship or family problems
- ☐ Have relationship or family breakdown
- ☐ Become dependent on alcohol, drugs or sedatives
- ☐ Lack self-care (e.g. have a change in their personal hygiene habits)
- ☐ Lose their job
- ☐ Have suicidal thoughts or behaviours
- ☐ Stop doing things they enjoy
- ☐ Develop new physical health problems
- ☐ Withdraw from close family and friends
- ☐ Don't know
- ☐ Other (please specify):_______________

**The word depression often means different things to different people. In the following questions, what we mean by "depression" is an illness that is more severe, more prolonged and more disabling than normal sadness, grief or other normal feelings of sadness or loss.**

Q14. What proportion of people do you think experience depression at some point in their lives?

- ☐ 1 in 50 people
- ☐ 1 in 20 people
- ☐ 1 in 10 people
- ☐ 1 in 5 people
- ☐ Don't know

Q15. What chance is there that you, or someone very close to you, will experience depression at some point in their lives?

- ☐ Zero to 25%
- ☐ 26 to 50%
- ☐ 51 to 75%
- ☐ 76 to 100%
- ☐ Don't know

**You can finish this survey here, but we would appreciate if you would complete the next section.**

## C. HELP AND TREATMENT

Q16. What would be the most likely result if you, or someone very close to you, received professional help for depression (e.g. from a doctor, psychologist, psychiatrist or other counsellor)? [mark 1 answer only]

- ☐ Fully recover
- ☐ Fully recover but then have the illness come back again
- ☐ Have some improvement
- ☐ Have some improvement but then get worse again
- ☐ Have no improvement
- ☐ Get worse
- ☐ Don't know
- ☐ Other (please specify): ____________________________

Q17. What would be the most likely result if you, or someone very close to you, did NOT receive professional help for depression ? [mark 1 answer only]

- ☐ Fully recover
- ☐ Fully recover but then have the illness come back again
- ☐ Have some improvement
- ☐ Have some improvement but then get worse again
- ☐ Have no improvement
- ☐ Get worse
- ☐ Don't know
- ☐ Other (please specify):____________________________

Q18a. If you thought you might be experiencing depression, how likely would you be to seek help from each of the following professionals ? [please rate all 9 categories]

| | Definitely unlikely | Probably unlikely | Probably likely | Definitely likely | Don't know |
|---|---|---|---|---|---|
| 1. Counsellor | ☐ | ☐ | ☐ | ☐ | ☐ |
| 2. General or family doctor | ☐ | ☐ | ☐ | ☐ | ☐ |
| 3. Pharmacist | ☐ | ☐ | ☐ | ☐ | ☐ |
| 4. Psychiatrist | ☐ | ☐ | ☐ | ☐ | ☐ |
| 5. Psychologist | ☐ | ☐ | ☐ | ☐ | ☐ |
| 6. Social worker | ☐ | ☐ | ☐ | ☐ | ☐ |
| 7. Welfare officer | ☐ | ☐ | ☐ | ☐ | ☐ |
| 8. No-one / wouldn't seek help | ☐ | ☐ | ☐ | ☐ | ☐ |
| 9. Other (please specify): ____________________ | ☐ | ☐ | ☐ | ☐ | ☐ |

Q18b. If you thought you might be experiencing depression, how likely would you be to seek help from each of the following people? [please rate all 8 categories]

| | Definitely unlikely | Probably unlikely | Probably likely | Definitely likely | Don't know |
|---|---|---|---|---|---|
| 1. Acupuncturist | ☐ | ☐ | ☐ | ☐ | ☐ |
| 2. Clergy, priest or other religious person | ☐ | ☐ | ☐ | ☐ | ☐ |
| 3. Family | ☐ | ☐ | ☐ | ☐ | ☐ |
| 4. Friends | ☐ | ☐ | ☐ | ☐ | ☐ |
| 5. Naturopath or herbalist | ☐ | ☐ | ☐ | ☐ | ☐ |
| 6. Personal trainer, exercise manager or relaxation instructor (e.g. massage therapist, yoga or meditation teacher) | ☐ | ☐ | ☐ | ☐ | ☐ |
| 7. Traditional healer (e.g. Qigong master, shaman) | ☐ | ☐ | ☐ | ☐ | ☐ |
| 8. Other (please specify): ____________________ | ☐ | ☐ | ☐ | ☐ | ☐ |

Q19. Do you think each of the following types of treatment for depression are helpful or harmful? [please rate all 11 categories]

| | Harmful | Neither | Helpful | Never heard of it | Don't know |
|---|---|---|---|---|---|
| 1. Becoming more physically active (e.g. playing sport, walking, gardening) | ☐ | ☐ | ☐ | ☐ | ☐ |
| 2. Changing your diet | ☐ | ☐ | ☐ | ☐ | ☐ |
| 3. Having an occasional alcoholic drink | ☐ | ☐ | ☐ | ☐ | ☐ |
| 4. Reading about people with similar problems and how they have dealt with them | ☐ | ☐ | ☐ | ☐ | ☐ |
| 5. Reading self-help book(s) | ☐ | ☐ | ☐ | ☐ | ☐ |
| 6. Taking antidepressant medications | ☐ | ☐ | ☐ | ☐ | ☐ |
| 7. Taking natural remedies (e.g. vitamins) | ☐ | ☐ | ☐ | ☐ | ☐ |
| 8. Taking sleeping tablets or sedatives | ☐ | ☐ | ☐ | ☐ | ☐ |
| 9. Using brief counselling therapies (eg. cognitive and/or behavioural therapies) | ☐ | ☐ | ☐ | ☐ | ☐ |
| 10. Using long-term counselling | ☐ | ☐ | ☐ | ☐ | ☐ |
| 11. Other (please specify):________________ | ☐ | ☐ | ☐ | ☐ | ☐ |

Q20a. Have you, or someone very close to you, ever experienced depression?

- ☐ Yes (please go to Q20b)
- ☐ No (go to section D)
- ☐ Don't know (go to section D)

Q20b. Who was that?

- ☐ I experienced depression
- ☐ Someone very close to me experienced depression

Q20c. Did you, or someone very close to you, receive any help for this?

- ☐ Yes (please go to Q20d)
- ☐ No (go to section D)
- ☐ Don't know (go to section D)

Q20d. Who provided this help (you can mark more than 1 answer)?

- ☐ Counsellor
- ☐ General or family doctor
- ☐ Pharmacist
- ☐ Psychiatrist
- ☐ Psychologist
- ☐ Social worker
- ☐ Welfare officer
- ☐ Don't know
- ☐ Other (please specify):________________________

Q20e. Did any of these other people provide help (you can mark more than 1 answer)?

- ☐ Acupuncturist
- ☐ Clergy, priest or other religious person
- ☐ Personal trainer or exercise manager
- ☐ Family
- ☐ Friends
- ☐ Naturopath or herbalist
- ☐ Relaxation instructor (e.g. massage therapist, yoga or meditation teacher)
- ☐ Traditional healer (e.g. Qigong master, shaman)
- ☐ Don't know
- ☐ Other (please specify):________________________

Q20f. Where did you or the person close to you receive help for depression?

- ☐ Specialist mental hospital
- ☐ General medical hospital
- ☐ General or family doctor's rooms / clinic
- ☐ Specialist doctor's rooms or clinic (i.e. psychiatrist)
- ☐ Other medical specialist doctor's rooms or clinic (e.g. neurologist, cardiologist)
- ☐ Other counsellor or therapist's rooms or clinic
- ☐ Clinic run by nurses or other health professionals
- ☐ Other (please specify):________________________

**You can finish this survey here, but we would appreciate if you would complete the next section.**

## D. INFORMATION

Q21a. Have you ever looked for information about depression?

- ☐ Yes
- ☐ No (go to section E)

Q21b. If YES, how did you get this information (you can mark more than 1 answer)?

- ☐ Asked a doctor
- ☐ Asked a friend
- ☐ Asked a family member
- ☐ Bought a book or health magazine
- ☐ Called a helpline
- ☐ Contacted a community health centre
- ☐ Contacted a mental health organisation
- ☐ Printed information from pharmacies or medical centre
- ☐ Searched the Internet
- ☐ Visited the library
- ☐ Television or radio
- ☐ Don't know
- ☐ Other (please specify):________________________

**You can finish this survey here, but we would appreciate if you would complete the next section.**

## E. PERCEIVED NEEDS

Q22a. Have you personally sought help from a general or family doctor for an emotional problem in the last 12 months?

☐ Yes ☐ No (go to section F)

Q22b. The following questions ask whether you would like your general or family doctor to discuss with you any of the following kinds of help for common emotional problems such as feeling depressed or anxious. Your general or family doctor might offer to help you in this way, or you might prefer your general or family doctor to suggest an alternative source of help.

| | | I would like my general or family doctor to discuss this kind of help with me | I don't need to discuss this kind of help | I am already getting this kind of help (either from my general or family doctor or somewhere else) |
|---|---|---|---|---|
| 1. | Information about emotional problems or getting treatment for these problems | ☐ | ☐ | ☐ |
| 2. | Medication or tablets to help you with emotional problems | ☐ | ☐ | ☐ |
| 3. | Counselling: including any kind of help to talk through your problems | ☐ | ☐ | ☐ |

Q22c. Have any of the following reasons stopped you in the last few weeks, from getting any of these kinds of help, or from getting as much help as you may have needed? Mark any box that applies to you:

☐ Not applicable, I haven't needed any of these kinds of help...

☐ I preferred to manage myself

☐ I didn't think anything would help

☐ I didn't know where to get help

☐ I was afraid to ask for help or what others would think of me

☐ I couldn't afford the money

☐ I asked but didn't get help

## F. ATTITUDES

**The following questions ask about the experiences people with mental illness sometimes have. Discrimination here means that a person with depression is treated unfairly just because they have a mental illness, rather than for any other reason.**

Q23. If you, or someone very close to you experienced depression, do you think you would be discriminated against by (please rate all 9 categories):

| | Definitely unlikely | Probably unlikely | Probably likely | Definitely likely | Don't know |
|---|---|---|---|---|---|
| 1. A bank, insurance company or other financial institution | ☐ | ☐ | ☐ | ☐ | ☐ |
| 2. A government or other public welfare agency | ☐ | ☐ | ☐ | ☐ | ☐ |
| 3. A public or private hospital | ☐ | ☐ | ☐ | ☐ | ☐ |
| 4. Other people who don't know you well | ☐ | ☐ | ☐ | ☐ | ☐ |
| 5. Your doctor or other health professional | ☐ | ☐ | ☐ | ☐ | ☐ |
| 6. Your employer | ☐ | ☐ | ☐ | ☐ | ☐ |
| 7. Your family | ☐ | ☐ | ☐ | ☐ | ☐ |
| 8. Your friends | ☐ | ☐ | ☐ | ☐ | ☐ |
| 9. Other (please specify):________________ | ☐ | ☐ | ☐ | ☐ | ☐ |

**The following question asks about people with severe depression. By severe depression we mean a depressive illness that is so extreme and distressing that the person may require specialised medical treatment, or the impact of the depression on their lives is very large (the person may not be able to work or socialise).**

Q24. To what extent do you agree or disagree with the following statements regarding people with severe depression? (please rate all 10 categories)

| **"People with severe depression …"** | Strongly disagree | Disagree | Agree | Strongly agree | Don't know |
|---|---|---|---|---|---|
| 1. Are dangerous to others | ☐ | ☐ | ☐ | ☐ | ☐ |
| 2. Are hard to talk to | ☐ | ☐ | ☐ | ☐ | ☐ |
| 3. Are often artistic or creative people when they are well | ☐ | ☐ | ☐ | ☐ | ☐ |
| 4. Are often very productive people when they are well | ☐ | ☐ | ☐ | ☐ | ☐ |
| 5. Have themselves to blame | ☐ | ☐ | ☐ | ☐ | ☐ |
| 6. Often make good employees when they are well | ☐ | ☐ | ☐ | ☐ | ☐ |
| 7. Often perform poorly as parents | ☐ | ☐ | ☐ | ☐ | ☐ |
| 8. Often try even harder to contribute to their families or work when they are well | ☐ | ☐ | ☐ | ☐ | ☐ |
| 9. Shouldn't have children in case they pass on the illness | ☐ | ☐ | ☐ | ☐ | ☐ |
| 10. Should pull themselves together | ☐ | ☐ | ☐ | ☐ | ☐ |

**You can finish this survey here, but we would appreciate if you would complete the next section.**

## G. GENERAL INFORMATION

**The following questions ask about how you have been feeling in recent times ...**

Q25. In the past 30 days how often (please rate all 10 categories) ...

| | None of the time | A little of the time | Some of the time | Most of the time | All of the time |
|---|---|---|---|---|---|
| 1. Did you feel tired out for no good reason | | | | | |
| 2. Did you feel nervous | | | | | |
| 3. Did you feel so nervous that nothing could calm you down | | | | | |
| 4. Did you feel hopeless | | | | | |
| 5. Did you feel restless or fidgety | | | | | |
| 6. Did you feel so restless that you could not sit still | | | | | |
| 7. Did you feel depressed | | | | | |
| 8. Did you feel that everything was an effort | | | | | |
| 9. Did you feel so sad that nothing could cheer you up | | | | | |
| 10. Did you feel worthless | | | | | |

Q26. Over the past few weeks have you been troubled by (please rate all 12 categories) ...

| | Never or some of the time | A good part of the time | Most of the time |
|---|---|---|---|
| 1. Feeling nervous or tense? | ☐ | ☐ | ☐ |
| 2. Muscle pain after activity? | ☐ | ☐ | ☐ |
| 3. Feeling unhappy and depressed? | ☐ | ☐ | ☐ |
| 4. Needing to sleep longer? | ☐ | ☐ | ☐ |
| 5. Prolonged tiredness after activity? | ☐ | ☐ | ☐ |
| 6. Feeling constantly under strain? | ☐ | ☐ | ☐ |
| 7. Poor sleep? | ☐ | ☐ | ☐ |
| 8. Everything getting on top of you? | ☐ | ☐ | ☐ |
| 9. Poor concentration? | ☐ | ☐ | ☐ |
| 10. Tired muscles after activity? | ☐ | ☐ | ☐ |
| 11. Losing confidence? | ☐ | ☐ | ☐ |
| 12. Being unable to overcome difficulties? | ☐ | ☐ | ☐ |

Q27. During the last one month:

a. How many days in total were you unable to carry out your usual daily activities, like going to work or school, fully?

☐☐ days

b. How many days in total did you stay in bed all or most of the day because of your illness or injury?

☐☐ days

Q28. Who do you live with?

- ☐ Live alone
- ☐ Live alone with child(ren)
- ☐ Live with partner and no child(ren)
- ☐ Live with partner and child(ren)
- ☐ Live with parents
- ☐ Live with other relatives
- ☐ Live with friends
- ☐ Live in shared accommodation
- ☐ Other (please specify):_______________________

Q29. Which of these best describes your main activities?

- ☐ Full-time work (including self-employed)
- ☐ Part-time work (including self-employed)
- ☐ Employed but not at work due to illness or vacation etc
- ☐ Not working and currently receiving sickness allowance or disability support pension
- ☐ Unemployed or looking for work
- ☐ Volunteer work
- ☐ Retired
- ☐ Home duties
- ☐ Student attending school or university
- ☐ Other (please specify):______________________________

Thank you for taking the time to complete this survey
